# ‘It was better when it was worse’: blue-collar narratives of the recent past in Belgrade

**DOI:** 10.1080/03071022.2018.1393997

**Published:** 2017-12-16

**Authors:** Rory Archer

**Affiliations:** ^a^ School of Slavonic and East European Studies, University College London, London, UK

**Keywords:** Belgrade, Serbia, Yugoslavia, working class, blue-collar workers, oral history

## Abstract

Based on oral history research conducted among networks of blue-collar workers in Belgrade, Serbia, this article develops three interrelated arguments regarding workers’ appraisals of the recent past (1980–2014). Firstly, although the tumultuous years of late socialism and post-socialism in Serbia have been represented by scholars as a series of ruptures, I suggest that for blue-collar workers the boundaries between socialism and post-socialism and pre-conflict and wartime eras are blurry. Secondly, despite the conditions of war and economic collapse, blue-collar accounts of the 1990s in Serbia are not universally negative. Some individuals experienced upward social mobility, strongly influenced by class and gender positioning in late socialism. Female workers who had experienced hardship during the 1980s were often better equipped to navigate 1990s ‘economies of makeshift’. Thirdly, social dislocation associated with neoliberal economic reforms since 2000 disproportionally affects blue-collar workers, reshaping narratives of late socialism and the 1990s (sometimes inducing workers to overlook or downplay coercive aspects of the Milošević regime). The accounts of this diverse group of (former) workers highlight that social class, gender and generational cohort condition the rather divergent ways in which the last three decades were experienced, are remembered and continue to be reevaluated in Serbia.

A graffito sprayed on a peeling wall and illuminated by the afternoon sun which pierces through the arches of Pula amphitheatre in the Croatian port city, declares ‘it was better when it was worse’ (see Figure 1). The expression originates from an Italian comic book series Maxmagnus (not as well known as the beloved Alan Ford but nonetheless quite popular in Yugoslavia). In issue 17 of the series, ‘After the Revolution’, a dissatisfied citizen daubed ‘it was better when it was worse’ on a city wall.^1^ The trope of it being ‘better when it was worse’ emerges rather frequently among blue-collar (former) Yugoslavs in discussions of the recent past, in particular when recalling personal experiences of macro events and processes like state dissolution, the dismantling of the socialist political and economic order, war, sanctions, unemployment and continued socioeconomic deprivation since the 2008 global financial crisis.

**Figure 1.**
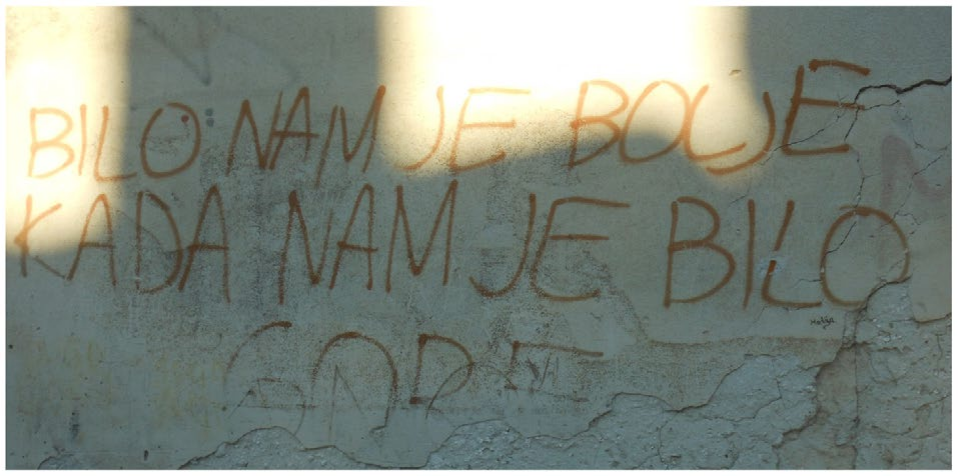
‘It was better when it was worse’. Graffiti in Pula, Croatia (photographed in 2014 by the author).

Ethnographic and narrative based scholarship of former Yugoslav societies has advanced the notion of a ‘before/after’ framework for understanding purported societal ruptures associated with violent conflict and state dissolution. Scholars including Stef Jansen, Jelena Obradović-Wochnik, Ljubica Spaskovska and Anders Stefansson have observed that interlocutors commonly invoke a ‘then/now’, ‘pre-war/post-war’ dichotomy when recalling the recent past.^2^ Obradović-Wochnik writes that ‘[w]hilst Serbia has moved on, politically and economically, most lives remain where they were interrupted in 1991’.^3^ Spaskovska claims in her historical research of youth subcultures in late socialism that the ‘outer contours of the Yugoslav chronotope’ are frequently marked by the outbreak of war for informants. ‘The Yugoslav space-time ends where the first bullets are fired, in the interval between mid-1991 and April 1992, depending on the physical location of the informant.’^4^

While acknowledging that such a framework may be salient for many individuals, this article argues that attributing a universal sense of rupture to post-socialist and post-conflict societies like Serbia demands some caution. By drawing on oral history research conducted among a cohort of blue-collar Belgraders I demonstrate that for most of these narrators, ruptures are multiple and temporally dislocated. Dichotomies like ‘before the war/after the war’, ‘then/now’, ‘in Yugoslavia/after Yugoslavia’ are subjective and surprisingly elastic. They are interlaced with and complicated by the socioeconomic upheavals associated with the crisis years of late socialism *and* the post-socialist era, in addition to the well documented disruptive impact of state dissolution and wars of the 1990s.^5^ Furthermore, the analysis of oral history narratives demonstrates that understandings of rupture tend to be conditioned by one’s social class, gender and generational cohort (among other factors).

Many academic and journalistic accounts of everyday life in Serbia in the 1990s accurately portray abysmal living conditions and stress the impoverishment of the middle classes. The devastation of war, sanctions and hyperinflation is frequently presented in pathological and amorphous terms by scholars and their informants alike as the apex of social deprivation.^6^ Obradović-Wochnik, in her study of Belgraders’ narratives about war and war crimes, writes that a notable feature of discussions on the decade is the conflation of negative events whereby ‘everything experienced at that time seems to have been lumped together in to one box of “really ugly memories”’ – wars, inflation, social deprivation and NATO airstrikes.^7^ Marko Živković’s anthropological study of Serbia in the 1990s similarly draws attention to murky and confusing representations of conditions.^8^ Yet, although dominant in academic accounts of 1990s Serbia, such portrayals are by no means universal. Some Belgraders were able to adjust to the new, unstable circumstances and achieve a modest degree of upward social mobility during the dire conditions of the 1990s. While few are likely to speak about the 1990s with unbridled enthusiasm, blanket criticism is not ubiquitous.

Like post-communist nostalgia, asserting that ‘it was better when it was worse’ in a post Yugoslav context serves as a means to critique the present and make particular socioeconomic and political claims in given social and historical circumstances.^9^ Yet, unlike nostalgia, it does not usually invoke a golden age or necessarily romanticize a bygone era. Instead, personal experience within different socioeconomic and political orders are compared in order to claim that ‘while it may have been bad then, it is worse now’ and so highlighting the sense of hopelessness and despondency that many workers and former workers in contemporary Serbia find themselves marred in.^10^ Blue-collar workers stress that they lived better in the recent past even though objective conditions may have appeared worse. Yet, rather than expressing a longing for either an idealized socialist past, support for the authoritarian regime of Slobodan Milošević in the 1990s or the anticipation of a ‘EU-ropean’ future, the narratives of blue-collar Belgraders tend to be more ambivalent and attest to the limits of attempting to impose immutable value categories among diverse social actors vis-à-vis the recent past.

## Methodological considerations: conducting oral history among blue-collar Belgraders

The goal of the oral history approach goes beyond mining oral sources for new empirical data. Historians like Luisa Passerini and Alessandro Portelli have convincingly demonstrated that oral history, as a ‘method of collecting narratives from individuals for the purpose of research’ is capable of fleshing out connections between individual biographical experience and the larger socio-historical context in which biographies are played out and so inform upon the *meaning* that events and processes held for individuals.^11^ Thus Portelli writes that oral history is capable of revealing ‘not just what people did, but what they wanted to do, what they believed they were doing, and what they now think they did’.^12^

In a Yugoslav context, oral history is well poised to capture previously neglected facets of social life, particularly given the liminal context observed by Spaskovska whereby ‘Yugoslav time is historical, while the (post)Yugoslav space and many people who inhabited that time and space are still in existence’.^13^ Historiography of late socialist Yugoslavia and its dissolution has relied on interviews with political and intellectual elites and community leaders.^14^ Oral history, however, has remained marginal – with the notable exception of Alvin Magid’s work on the legitimacy of the Yugoslav system, more recent research probing the memories of Yugoslav socialism among female textile workers by Chiara Bonfiglioli and Nina Vodopivec, and a study of cable factory workers in Serbia by Tanja Petrović.^15^ Yugoslav sociological and historical enquiry tended towards quantitative and empiricist forms and consequently very little research explores the lives of blue-collar workers in a qualitative sense.^16^

In the historiography of other socialist and post-socialist societies in Eastern Europe and the (former) Soviet Union, oral history approaches are more developed and theoretically informed. A number of ambitious longitudinal studies make use of oral history narratives like Michael Westrate’s extensive study of Kharhiv ‘from Stalin to Maidan’, Miroslav Vanek’s monograph on Czech society, and an edited volume *On living through Soviet Russia*.^17^ Other works hone in on particular themes in detail, including generational cohorts like the Soviet baby boomers, communal apartment living and traumatic accounts of gulag survivors.^18^ Oral history research also tackles the gendered nature of socialism and the particular experiences of women in Czechoslovakia, Bulgaria and elsewhere.^19^ As well as grappling with the lived experience of socialism and post-socialist transformations much of this body of work (like the recent volume edited by Khanenko-Friesen and G. Grinchenko) is in close dialogue with critical developments in the field of oral history in terms of theory and method.^20^ Yugoslavia and its successor states on the other hand, remain outliers in such discussions.

In this study, I draw upon open-ended life history interviews conducted with 25 blue-collar residents of Belgrade during 2014. Undertaken as part of a larger project on labour and everyday life in late socialist Yugoslavia, the oral history research was combined with the analysis of print media and archival documents relating to the particular workplace and local community.^21^ The interviews centred on the workplace and (non) access to housing in Yugoslavia but narrators were encouraged to speak about their lives in broader terms. Keeping in mind Trevor Lummis’s distinction between ‘memory’ and ‘recall’, the focus on the workplace and housing is utilized as a methodological tool to generate ‘responses to detailed interviewing which prompts dormant “memories” that are less likely to be integrated into the individual’s present value structure’ (as opposed to more polished structures which suggest the narrative has been retold or thought about).^22^ This relies on the assumption that many individuals encountered difficulties in accessing housing and employment which they could accurately recall and link to broader trends in their workplace, local community and society. Narrators with whom I spoke recall housing being a particularly controversial and topical issue in the workplace around which issues of class, privilege and socialist morality crystallized.^23^

All narrators were living in Belgrade permanently in 2014 and resided in the municipalities of Čukarica, Grocka, Novi Beograd, Rakovica and Zvezdara and in the nearby towns of Mladenovac and Lazarevac (both administered as municipalities of Belgrade). While the social structure of these municipalities is quite diverse (ranging from upmarket, centrally located apartment complexes in Novi Belgrade to subpar informal settlements in the peripheries of Rakovica) they do encompass many of Belgrade’s industrial, working-class suburbs. No narrators lived in the downtown municipality of Stari Grad, the upmarket Savski Venac encompassing the villas of Dedinje and Senjak, nor in Vračar, a well-heeled residential hub of the pre-socialist and socialist bourgeoisie.

Belgrade is a city which has been shaped by large-scale inward migration from other parts of Serbia and Yugoslavia during the twentieth century. The influx of rural migrants caused the population to double from 1944 to 1962 with Belgrade approaching a million inhabitants by 1969.^24^ The city’s population reached 1.5 million by 1981 and almost 1.7 million by the end of the decade.^25^ Rural to urban migration has been a perennial source of trepidation and even outright hostility for some Belgraders who symbolically draw upon an urbane, city identity through a discourse that ‘others’ newcomers to the city – firstly rural migrants and subsequently refugees during the 1990s.^26^ In his seminal study of rural-to-urban migration in 1960s Belgrade, Andrei Simić argues that the city was a ‘funnel for the diffusion of traits from the outside world’ while simultaneously being the subject of further ‘peseantization’ and ‘Serbianization’; a consequence of inward migration.^27^ Like Simić I consider that Belgrade is ‘the optimal site in [former] Yugoslavia for investigating the effects of social and spatial mobility, and the resultant dynamics of acculturation and culture change’.^28^

The non-Belgrade background of many contemporary Belgraders is an indicative feature of its populace and the small sample of oral history narrators seeks to reflect this diversity. When conducting research, I decided that instead of disqualifying potential narrators because of non-Belgrade origins, I would instead establish their residency patterns and try to ensure that some of the historically representative migration paths were captured in the sample in addition to the narratives of born-and-bred Belgraders. Approximately one third of narrators were born in the city and grew up in Belgrade and its environs. Another third came to Belgrade by early adulthood (over two thirds of narrators were resident in Belgrade by 1980) while the remaining third moved to the city permanently between 1980 and 2002 as adults. All narrators had lived in Belgrade for more than a decade prior to the interviews taking place and the city was their primary frame of reference. In addition to Serbia, narrators were born in Bosnia and Herzegovina, Croatia, Montenegro and the autonomous provinces of Vojvodina and Kosovo. Thus as well as reflecting upon conditions in greater Belgrade some narrators also spoke about living and working in Osijek, Rijeka, Zadar, Knin, Vukovar, Prizren, Nikšić, Smederevo, Subotica and Novi Sad, giving a pan-Yugoslav perspective to the narratives.

I made contact with narrators through networks of friends, acquaintances and colleagues during a year of field research in Belgrade. Narrators then introduced me to their colleagues, family members and neighbours. I began interviews with a set of open-ended biographical questions (‘where were you born? where did you grow up?’). I did not wish to impose a particular narrative framework – narrators were not interrupted from their particular narrative frame once they began speaking. However, being able to anchor the interview in terms of life events such as entering the workforce and accessing housing enabled me to return to a concrete time period should the interview go wildly off-topic. Interviews were conducted in the narrator’s home (or that of their neighbour) in Serbia and have been anonymized through the use of pseudonyms. I came to realize that the most important factor in establishing a collaborative rapport with narrators was their ability to ‘locate’ me in their social space – usually in relation to the contact that arranged our meeting (‘that foreign friend of Miloš who speaks Serbian’, ‘That Irish boy the Jovanovićs spoke to yesterday’). A couple of narrators openly expressed that they would not give an interview to somebody without some form of personal contact (let alone let that person into their home). My ‘outsiderhood’ as a foreign researcher prompted unsolicited and solicited clarifications of language and terminology during interviews providing further insight into how narrators conceived of (or at least verbalized) various phenomena.^29^

Participants were not directly asked to speak about the recent past or to appraise it in an abstract sense but rather to discuss their own lives in subjective terms (their own memories of access to employment and housing) which unfolded in the socio-political context of late socialism in the 1970s and 1980s and the collapse of the Yugoslav state in the 1990s. I did not explicitly request that narrators state their religion or ethnonational affiliation but gauged from discussions that most identified as ethnic Serbs and a significant number were practising Orthodox Christians (neither of which precluded a pro-Yugoslav orientation or communist sympathies). A couple of individuals claimed to be ‘not really believers’ if not quite fully fledged atheists and one family from Kosovo identified themselves as Roma members of the Serbian Orthodox Church. Despite the predominance of ethnonationalism in the public domains of former Yugoslav states, ethnicity was not a category that held great sway for most narrators in terms of narrating experiences of socioeconomic change *in the realm of their everyday lives*. This is not to claim that this cohort of workers held explicitly antinationalist political orientations – while a few did express such views the majority of narrators tended to conceive of nations and nationalisms in essentialist terms. Nevertheless, national categories or discourse that might be framed as nationalist ideology were not usually drawn upon by blue-collar workers when reflecting on their subjective experience of change between the 1980s and 2010s. Thus I am in agreement with V.P. Gagnon and social anthropologists working in the former Yugoslavia in acknowledging the salience of ethnonationalism while at the same time recognizing the limits of employing ethnonational paradigms in the analysis of social relations at the micro, everyday level.^30^

## The Yugoslav working class in the crisis years of late socialism

The categorization of ‘working class’ or ‘blue-collar worker’ in any social context is not unproblematic and Yugoslavia is no exception. The ambivalence of official Yugoslav conceptualizations of class is belied by the terminology deployed by state authorities. In official discourse the term ‘working people’ (*radni ljudi* or *radni narod*) was used alongside ‘working class’ (*radnička klasa*) to symbolically incorporate non-industrial workers into the body politic. Most partisan fighters during World War Two hailed from peasant origins and despite the rapid pace of industrialization and urbanization after the war, a significant agrarian population remained *in situ*. The mixing of these categories can be seen for example in the Yugoslav constitution of 1974 which proclaimed that ‘In the Socialist Federative Republic of Yugoslavia all power belongs to the working class in alliance with all working people of the town and village’ (giving symbolic weight to rural as well as urban workers as well as to employees in non-productive settings).^31^ The Yugoslav working class was both a value category informed by Marxist-Leninist ideology as well as a social reality brought about by industrialization and urbanization. Yugoslav social research struck a balance between ideological and sociologically informed conceptualizations. From the late 1960s issues pertaining to social inequalities, stratification and class could be legitimately discussed and became a key research interest of many Yugoslav social scientists.^32^

I conducted interviews with 25 individuals of a working-class background which was defined for selection purposes as those who had worked in direct production (i.e. on the shop floor) in Yugoslav factories at some point during the 1970s and 1980s. The social position of workers was often contradictory in practice and changed over time; it was influenced by the skills in demands in the particular economic sector, the position of the firm on the self-managing Yugoslav market and local conditions in a period when decentralization and autarky were on the ascent.^33^ Sometimes workers in direct production considered themselves privileged due to what they recall as comparatively high wages supplemented by bonuses for exceeding norms. Yet at other times these same workers considered themselves de-privileged. They cited the risk of injury (or injuries which were actually sustained), long hours and wage contractions as inducing them to leave direct production and seek a position elsewhere in the company or in another firm. Some narrators thus worked for a shorter time in blue-collar positions before continuing adult education and transferring to administrative positions like typing and bookkeeping or janitorial work (with pregnancy, illness or a workplace injury often precipitating such a transfer). Moving to such a position was not necessarily accompanied by a commensurate increase in pay or prestige. Feminized positions such as cleaning and typing were typically devalued in the workerist hierarchy of labour and so were accordingly poorly paid. Even among the small sample of narrators informing this text, attempts to differentiate between blue- and white-collar workers is fraught with difficulty with many individuals moving between these roles during their working lives.

An observable trend which affected blue-collar workers more intensely than white-collars, however, was decreasing wages and falling living standards in the 1980s. The oil crisis of the late 1970s accentuated Yugoslavia’s economic problems, in particular foreign debt which approached 20 billion US dollars by the early 1980s. The country entered recession in 1979, the same year as Edvard Kardelj’s death. Josip Broz Tito died in 1980 prompting a mass outpouring of grief (as well as a certain degree of apprehension about the future, particularly the sustainability of the rotating presidency system). The consequences of the post 1979 economic crisis and austerity measures were not socially neutral. Pre-existing social and regional inequalities which had been sharply rising since the 1960s were aggravated.^34^ From 1981 the country committed to enforcing programmes of export driven economic ‘stabilization’ to repay foreign debt. In 1983 the Federal Executive Council accepted the ‘Long-Term Programme for Economic Stabilisation’ which affirmed a liberalised market-based economy and austerity measures under the continued tutelage of the League of Communists of Yugoslavia without displacing the 1976 Law on Associated Labour or other components of self-management.^35^ Susan Woodward writes that ‘stabilisation’ sought to ‘reorient domestic institutions to Western markets and foreign price competition (“integration into the international division of labor”) and to increase productivity in manufacturing, again by technological modernization through imports’.^36^ A knock-on effect of stabilization, however, was a massive drop in living standards which disproportionally affected blue-collar workers. Branka Magaš, writing in 1989, pointed out that the crisis ‘did not affect all social layers equally’ and ‘hit the working class with special severity as industrial growth stopped or went into reverse’.^37^ Registered unemployment, already 13.8% in the late 1970s reached 16.3% by 1985. Earnings fell by over 25% in the same period and aggregate inflation exceeded 1000%.^38^ By the mid-1980s some 40% of social sector workers were estimated to be living on the poverty line and pensions contracted by more than 40%.^39^ Living standards in the 1980s were pushed back to those of the 1960s with blue-collar workers affected most gravely.^40^ The gains of the 1970s, the decade which encapsulated the heyday of Yugoslav consumerism, had been virtually erased for many workers.^41^

At the same time, the Yugoslav working class was becoming ever more decoupled from the League of Communists and the formal institutions of self-management. Strikes increased dramatically in numbers and intensity throughout the 1980s, doubling every year between 1980 and 1987.^42^ By the end of the decade the per capita rate of strikes in Yugoslavia was the highest in Europe.^43^ Working-class subjectivities were not only being shaped in the workplace or through industrial action but also in ever more socially segregated urban spaces. Bogdan Denitch observed in the late 1980s that class solidarity fostered in the workplace among Yugoslav blue-collar workers was reinforced in increasingly segregated residential neighbourhoods leading to an ‘us and them’ attitude pitting workers against the communist politocracy and its technocrat allies.^44^ This article will now explore how Belgrade workers found themselves increasingly alienated vis-à-vis workplace management in the mid-1980s and how they relate this to the crisis and eventual violent dissolution of the country in the early 1990s.

## The destruction of sociability in the 1980s workplace

Many Belgrade-born narrators recalled a creeping ‘destruction of sociability’ in the workplace from about 1985 characterized by theft which increased in scale through the late 1980s and peaked in the 1990s. Rather than being viewed as the consequence of the break in the system after 1990, this is commonly understood on a continuum beginning in the 1980s in the context of economic crisis. The phrase ‘destruction of sociability’ is borrowed from Eric Gordy’s *The Culture of Power in Serbia* in which he attributes the durability of Milošević’s regime in the 1990s to the calculated destruction of political, media and musical alternatives.^45^ In drawing on this expression I suggest that the proliferation of difficulties like increasing workplace corruption, absenteeism, theft and unemployment in 1980s Yugoslavia, which were taking place against a backdrop of the ‘withering away of the state’ and austerity measures, can be considered as a precursor to post-socialism. By this I mean to stress that the socioeconomic difficulties associated with the early 1990s have a precedent in the 1980s and are understood as such by many blue-collar workers. The following examples serve to illustrate the process by which individuals relate negative aspects of their workplace in the 1980s to broader transformations which culminated in state dissolution in the early 1990s and an even more wide-ranging destruction of sociability in the context of war, sanctions and isolation.

Marija is a former worker at ‘Kvarc’, a manufacturer of materials for the metal industry in Mladenovac, a town less than an hour’s drive southeast of Belgrade. She began working in the factory in 1969, initially on the shop floor, then as a shift leader and later moved to the accounts department (making the transition from blue-collar to white-collar worker in the process). Marija elaborates on the workplace theft of the 1990s against the backdrop of war and socioeconomic dislocation in Milošević’s Serbia.

When this craziness began, war and all that, then the general theft of firms started, there was no control left […] It reached abnormal limits. I had colleagues who worked in purchasing who would do things like […] you calculate the goods more expensive by 50%, you take 20%, they take 30% […] cash-in-hand. When people increasingly worked with those private businesses [*sa tim privatnicima*] they made false prices to jointly steal from the firms. There were no longer limits. Workers lost all power, all influence then.^46^

However, rather than understanding mass theft and the waning of workers’ influence as a sudden consequence of the violent dissolution of the state and system, Marija traces its origins to the mid-1980s. She claims it was in this period that the influence of workers in the system began to falter. She described how colleagues (usually white-collar workers) were increasingly defrauding the workplace. For example, they would routinely enjoy pricey business lunches, arrange a grossly inflated forged bill from the waiter, and then pocket the difference.

This began in 1985, 1986, 1987 […] when it began to get shaky’ [… Theft and corruption] was then within the frame of the firm […] but after 1985, 1986 […] there was no longer control […] first in the frame of their workplace, all kinds of privilege, everything could just be taken…^47^

Individual workers were likely to condone minor theft according to a moral economy of real socialism which reacted to the ‘structural constraints of the socialist system of distribution’.^48^ However, with equal measure they condemned the abuses of managers within the faltering workplaces of the 1980s and 1990s. It was managers whose activities were seen not only as illegitimate but deeply damaging, far beyond the acceptable limits of the privatization of socially owned property. Drawing upon the concept of moral economy as developed by E.P. Thompson and later popularized in social anthropology by James Scott, Michael Lebowitz details a moral economy in real socialism.^49^ He maintains that such a moral economy was not a remnant of traditional peasant societies antecedent to state socialism but rather emerged as part of a novel social contract in which workers enjoyed job security and improvements in their living conditions in return for acquiescence in the political sphere.^50^ In cases where one’s workplace put the worker in contact with scarce material resources it was considered acceptable that individuals would make use of these resources for family and friends. Workers felt ‘very comfortable’ pilfering items as it was collective property and, so, partly theirs.^51^ Yet by the same token workers condemned large-scale theft of social property by management as part of heightened populist stirrings against the bureaucracy in Yugoslavia during the 1980s.^52^ Indeed the idea that white-collar workers and bureaucrats were enjoying privileges at the expense of the living standards of blue-collar workers was the fundamental grievance of Yugoslav workers during the 1980s.^53^

Marija’s recently acquired white-collar position put her in daily contact with ‘bureaucrats’ and management and thus she had a direct view of machinations taking place in her workplace and the municipality. Blue-collar narrators, however, also stressed the links between the political crisis of late socialism and the perceived increase in theft and corruption in the workplace and could recall numerous examples they had personally encountered. In the workers’ settlement of Makiš – a scattering of temporary barracks now converted into permanent workers’ homes trailing the perimeter of a long defunct wood processing plant on Belgrade’s southern fringes – I spoke with a group of neighbours about their experience of the 1980s workplace. Mirko, a retired electrician, when asked if he noticed the crisis in the 1980s responded that it was evident after Tito’s death in 1980 in a political sense (‘first one president, then eight of them, collective leadership…’).^54^ His neighbour Milovan interjects, ‘It [crisis] did not begin then, but in 1985’.^55^

Mirjana, Milovan’s wife, and her sister Gordana elaborate on this in more detail. Mirjana was a blue-collar worker at the local wood processing plant in Makiš for 12 years. Following in the footsteps of her mother, aunt and older sister, she began work in the collective as a cleaner and courier, later moving to the shop floor. Dissatisfied with the way the plant was being managed, in 1985 she gave her notice and left Makiš to take a job at a successful Belgrade textile factory, Kluz. She describes how she ‘saw it all deteriorating’ in Makiš as the management engaged in schemes to ever more blatantly defraud the company. In discussion with her sister Gordana, she describes theft on the part of management in the 1980s:

… in 1985, when I left, I knew it would fall apart. Because mass theft began. First the directors, can I say his name? I don’t care – [*Mirjana names the director*]. I worked as a courier then. He replaced materials. For example, first class materials should have been ordered but he would organize that third class materials come from Bosnia instead and he would pocket the difference. He was the technical director. The General Director […] said ‘how is it possible, what did you do to get third class materials?’ They did not notice that I was there bringing them coffee hearing the whole conversation! He replied ‘it was not me’ and the general director says ‘show me how to do it’. You could already feel then that it [the factory] was all going to collapse as soon as things like that were happening. Third class materials […] that is not quality, you cannot make windows and doors with that! Then, other kinds of theft began, you could feel it after Tito that it was all going to fall apart. This country […] After Tito, people started to steal massively.^56^

Mirjana recalls another manager who stole food vouchers for workers’ hot meals over a period of months and allegedly bought a flat with the proceeds. Mirjana and Gordana stressed that such machinations could only occur with the acquiescence of other managers in the firm and were far beyond the limits of pilfering that might be deemed acceptable among the blue-collar workers. Ultimately the manager in question was removed from his position and the theft was reported in print media. He was not prosecuted, however, and Gordana recalls that he took up work at another firm: ‘That’s how it went […] from when Tito died, it was terrible how it went [with corrupt directors]. They arranged work for one another, money laundering […] it’s like that today’.^57^

For narrators like sisters Gordana and Mirjana in Makiš and Marija in Mladenovac, blatant workplace theft on the part of management was not inherent to Yugoslav workers’ self-management but rather indicative of the rupture in that very system which became palpable by the mid-1980s. Although narrators acknowledged that a degree of theft, corruption and social inequality were perennial features of the Yugoslav workplace, they stress that from the mid-1980s onwards this took a more pathological form and thus contravened a moral economy according to which workers (blue-collar and white-collar alike) would help themselves to benefits-in-kind within acceptable limits. Rather than considering the break-up of the Yugoslav state in 1991 and the outbreak of war in Slovenia and Croatia that same year as the most significant rupture in their community, Belgrade-born workers like Marija, Gordana and Mirjana stressed the break with hitherto practice in the years following Tito’s death as of paramount importance and usually described the difficulties of the 1980s and 1990s on a single continuum, jumping back and forth between the socialist and post-socialist eras in one swoop.

I am in agreement with scholars cited in the introduction to this article that ‘before/after’ and ‘then/now’ are extremely resonant frameworks used by social actors to interpret major social ruptures in former Yugoslavia. However, in oral history interviews with blue-collar Belgraders it became clear that the actual *point* of rupture is not fixed and is usually not even internally consistent in an individual’s narrative. The framing is more flexible and ambiguous than a socialist/post-socialist or pre-war/wartime dichotomy suggests. Categorizations of ‘socialism’ and ‘post-socialism’, ‘before the war’ and ‘after the war’, are messy, often contradictory and deeply intertwined. Tanja Petrović’s claim that for workers in a central Serbian cable factory the ‘rigid dichotomy’ implied by the discourse of ‘European democracy’ and ‘East European Socialism’ was by no means salient also rings true for Belgrade workers in 2014.^58^ Ruptures associated with the collapse of the socialist order and dissolution of the Yugoslav state were manifold, shaped by one’s social position, proximity to conflict zones and experience of life in Yugoslavia before and during the crisis of late socialism. The negative phenomena associated with post-socialist dislocation (like the deterioration of social relations in the workplace and an increase in theft, absenteeism and corruption) became palpable to many narrators in the 1980s and thus sometimes the last years of socialism (the 1980s) are attributed the negative characteristics of 1990s post-socialist Serbia as the accounts of narrators like Marija, Mirjana and Gordana attest to. For communities more directly affected by war in the 1990s, however, the dynamics differ significantly and severe ruptures were experienced, as my discussion will now show.

## Conflict and rupture

Catherine Baker writes that it is ‘deeply rewarding’ to combine the analytical lenses of post-socialism and ethnopolitical conflict in relation to contemporary Bosnia Herzegovina.^59^ One might also productively investigate social phenomena in Serbia according to these parameters; as a site where one should take into account both post-socialist *and* post-conflict transformations. However, discussing Serbia in the context of being a ‘post-conflict society’ requires some qualification. Although the Republic of Serbia was deeply implicated in the Yugoslav wars of the 1990s, until the war in Kosovo and subsequent NATO bombing in 1999 open warfare did not occur on its territory (with the exception of Kosovo).

Nevertheless, Serbia was a heavily militarized society throughout the decade run by a coercive state apparatus.^60^ Serbian males were conscripted to the Yugoslav Army between 1991 and 1992 to fight in Slovenia and Croatia and again to fight in Kosovo in 1998 and 1999. Bosnian Serbs were transferred to Bosnia to fight with the nascent Army of Republika Srpska from 1992. Others volunteered in paramilitary formations throughout the 1990s and Serbia remained isolated under regimes of international sanctions for most of the decade. Belgrade was home to many (mostly but not exclusively Serb) refugees as well as former combatants and civilian participants of war in Bosnia, Croatia and Kosovo who escaped or migrated to the city. As a significant number of current Belgrade residents come from war-affected areas and all who were resident in the city during the 1990s experienced privations which were inextricably linked to the wars Serbia was waging across the former Yugoslavia, and later NATO airstrikes, one may to this extent consider Serbia to be a post-conflict society. However, the *meaning* of conflict and its legacies probably differs significantly from that of Bosnia, Kosovo or Croatia.

The topic of the wars of the 1990s predictably surfaced most prominently in discussions with narrators who had lived in locations affected by war – two narrators were from Prizren, Kosovo, and two narrators were born in Croatia (Knin and Western Slavonia). Other narrators had family ties to various parts of the former Yugoslavia and gathered both formal knowledge about conflict (from various state, independent and international media) and informal knowledge (through rumour and hearsay).^61^ In contrast to narrators like Mirjana, Gordana and Marija who claimed a systemic rupture in the mid-1980s when they were living and working in Belgrade, narrators from areas directly affected by conflict in the 1990s recalled severe ruptures prompted primarily by war.

Yet for narrators from conflict affected areas, ruptures in everyday life are far more varied than the 1991 outbreak of war and highly dependent on local conditions. Saša, a self-identified Serbian Orthodox Rom from Prizren, Kosovo’s second largest city, now a refugee living in Makiš, Belgrade, illustrates the context dependent nature in which conflict was experienced. For him and his family, their personal experience of rupture occurred upon his fleeing from Kosovo in June 1999 with most of Prizren’s Serb population. Until then Saša had lived in Prizren and worked as a blue-collar worker in a brick factory in the nearby town of Suva Reka. Conditions in Kosovo deteriorated throughout the 1980s and even more so after 1989 with the revocation of Kosovo’s autonomy.^62^ Saša recalls that after 1990, following the mass dismissal of Albanian workers, around 950 Serbs, over 20 Muslims (Bosniaks) and ‘a few Orthodox Roma’ (as he describes himself) were employed in the factory. Throughout 1998 and early 1999, as clashes between Serb armed forces and the Kosovo Liberation Army descended into war, Saša continued to go to work with his colleagues on buses with armed guards. Saša, perhaps somewhat incredulously given the violent, militarized conditions in his homeland, recalls the decade up to June 1999 with nostalgia.

*Šiptari* [*sic.*, derogatory term for Albanians] were in production previously, 2,500 of them. But we with 1,000 workers worked better. My soul still hurts for that. We were all young. When we were on the bus to work it was like we were going on some *akcija* [socialist youth working brigade], joking around, having fun [….] All young, let’s go to a café after work and so on. Ten years flew like that. There were friendships, romances […] marriages, *kumstvo*…^63^

For narrators like Saša who fled their home communities as refugees, war is represented as the most dominant rupture in their recent life trajectory. However, these ruptures may be temporally disparate and tend to relate to particular local conditions for one’s local community rather than for the (former) state as a whole. Thus rather than positing 1991 as a universal and logical point of rupture (the breakdown of the Yugoslav state and descent to war) narrators also cite alternate periods – 1992, 1995, 1998, 1999 – as particular ruptures they experienced in their community due to war.

## Subjective experiences of precariousness during late socialism and after

Conducting oral history work with individuals from divergent backgrounds highlights how experiences of post-socialist rupture are informed by the social position one held in Yugoslavia (permutations of social class, gender and generational cohort). Keeping this in mind the next section explores an account of the ‘economies of makeshift’ and upward social mobility which do not necessarily cohere with dominant representations of the former socialist elites and those of the Belgrade middle classes whose views have been prioritized in academic accounts of everyday life in Serbia during the 1990s. The following assertion of a Serbian academic from the mid-1990s is representative of such a view:

In the previous time [socialism] we lived an easy life, not on a high standard, but somehow, everything was easy – to go on holiday, to get a flat from the institution where you worked, to buy new clothes, to eat whatever you wanted, to have fun, to visit restaurants, to travel abroad, to have free medical care. Now we spend practically all our earned money only for food. Our clothes and shoes, as well as our health and good moods, come from the previous time. The winter of 1993/1994 was the hardest in my life. We lived by eating only potatoes and beans and we had to spend our life savings to buy that.^64^

Unlike most of the working-class oral history narrators, this individual considered that ‘everything was easy’ under socialism. Receiving a flat from one’s employer and engaging in various forms of leisure and consumption (clothes shopping, eating at restaurants regularly, foreign travel) were part of the ‘normal life course’ for their social milieu. Clearly this experience is indicative of a rather privileged social position – employment in an academic institution. Despite the authoritative ‘we’ used by the speaker, such conditions were not applicable to most of the working class employed in Yugoslav social sector workplaces, many of whom spent their working lives battling to access suitable housing and in the crisis years of the 1980s spending up to 70% of their wages on basic food supplies.^65^

In the following section I suggest that some individuals who lived as precarious workers in poor material conditions during the 1980s were better equipped to improve their social position after 1990 as they had gained practical experience – a particular kind of social capital – enabling them to operate beyond the state and self-managing system to enhance their own well-being. In other words, the experience of precariousness within late Yugoslav socialism induced some blue-collar workers to engage in an ‘economy of makeshifts’ which equipped them with the social capital necessary to navigate the dire economic conditions of the 1990s. The concept of a makeshift economy was initially developed by Olwen Hufton with regard to the subsistence strategies of the poor in eighteenth-century France.^66^ Since Hufton’s conceptualization, scholars have deployed makeshift economics in diverse contexts – from studies of poverty in eighteenth- and nineteenth-century English towns to homelessness in deindustrializing New York City of the 1980s.^67^ A Yugoslav ‘economy of makeshifts’ shares common features with earlier conceptualizations (though in the conditions of late-socialism religious charities were predictably not prominent). Ad-hoc solutions for Yugoslavia’s poor, (in parlance of the time ‘social cases’) would be procured through workplace trade unions, at municipal centres for social work or in local community organizations (*mesne zajednice*).^68^ A further feature of the Yugoslav economy of makeshifts was a reliance on workplaces for benefits-in-kind and a modicum of social security which could then be coupled with riskier but more lucrative entrepreneurial activities.

The parallel accounts of narrators Ljiljana and Nebojša highlight some varying generational, gender and class informed accounts of life in the 1980s and 1990s which help account for participation in a makeshift economy. Ljiljana lived in considerable hardship upon moving to Belgrade in 1978 from Western Serbia. She and her family spent nearly five years in a ‘miserable rented room’ before squatting in a block of flats in 1984 in Novi Beograd with her children and former husband, renovating a common laundry area which they converted in to a small flat. From 1986, Ljiljana began smuggling clothes from Turkey, Italy, Czechoslovakia and Germany to resell informally in her workplace, a large tractor and engine producer in Belgrade where she worked as a cleaner, courier and later on the shop-floor.

I would go to work and bring a big bag with t-shirts, pants, other outfits, and sold them to the workers. I earned good money from it […] I would charge two to three times as much as I had bought them for there. And that is the way I lived a bit more easily.^69^

Such undertakings, while illegal, were rather casual and punishment by authorities at state borders or the workplace was not too frightening a prospect. Ljiljana describes travelling to Italy by car and crossing the border multiple times with modest quantities of goods while a collaborator, usually her daughter, waited in the forest on the Yugoslav side of the border with the stash of goods. In this way she managed to smuggle ‘one or two thousand socks’. Her accounts cohere with other narratives of smuggling in Trieste in that she would often wear items of clothing across the border and somehow ‘get by’ when it came to encounters with customs officers who she recalls often tacitly acknowledged her smuggling endeavours (‘a bit of joking, smiling and you get through [the border]’).^70^ Selling goods like this in Belgrade workplaces was no rarity. A light hearted comment in the workplace periodical of Belgrade engine manufacturer ‘21. maj’ in 1980 suggested that this was a common occupation of female coffee makers like Ljiljana who were selling ‘better goods than at the flea market’ and bringing the flavour of Ponte Rosso (a popular shopping area of Trieste) into the Belgrade workplace.^71^

Due to this informal business, Ljiljana recalls that her personal financial situation stabilized in the late 1980s. This was in marked contrast to socioeconomic trends of the era; wage cuts of up to 40% and price increases of between 30% and 100% were being reported in Yugoslav newspapers.^72^ ‘There was no longer any big crisis for me and the children, to survive… to buy clothes and eat. But from the pay check alone it was always hard to survive.’^73^ As a result of her successful experience in smuggling clothes in the late 1980s Ljiljana continued to smuggle and diversified operations in response to the changing economic context, becoming the family breadwinner.

With the imposition of international sanctions on Serbia and Montenegro in 1992 ‘sanctions busting’ became an important factor of economic survival for both individuals (to receive hard currency for goods) as well as for the state (as a means to access essential goods and deflect social unrest).^74^ Peter Andreas stresses that although those closest to the Milošević regime reaped the greatest profits in smuggling, ‘sanctions also helped prompt broader participation in and tolerance of the underground economy’.^75^ ‘Sanctions busting’ was not only the preserve of a criminal elite and their cronies but also of enterprising individuals who sought to improve their standard of living. Ljiljana was well placed to engage in smuggling in this context and switched from informally selling Italian clothes in her workplace to hawking fuel on the streets of Belgrade, the import of which was restricted by the international sanctions regime. In 1993 she started smuggling petrol and diesel from Bulgaria to Serbia with her son regularly. Additionally, she queued overnight in front of West European embassies in Belgrade and sold the spot in the morning for up to 50 Deutsch Marks to visa applicants (usually to Serbian seasonal workers going to Greece or to Kosovo Albanians fleeing to Germany).

In contrast to accounts which describe the winter of 1993/1994 as amongst the most brutal for middle-class Belgraders (like the academic quoted earlier), Ljiljana maintains that despite the real value of her factory wage being reduced to a single Deutsch Mark due to hyperinflation, she did not feel the crisis that badly because she worked extensively in the grey economy:

Whoever knew how to get by, they got by [*Ko je znao da se snadje… kako se ko snalazio*] … I would go home [after smuggling or selling embassy spots], on the way I would stop by the market and buy my children *everything* so that they would have enough to eat and not feel the crisis.^76^

Furthermore, access to hard currency meant that Ljiljana could access credit in dinars, the repayments of which would be reduced to a meaningless sum by hyperinflation. Through her factory work she retained a modicum of social security – access to healthcare and pension contributions – while her informal jobs enabled her to raise enough hard currency from which to live. Although Ljiljana’s embodied labour was demanding physically, she recalls with proud satisfaction of having secured a living for her family in difficult times. Like the protagonists in Kristen Ghodsee’s study of Bulgarian female tourist workers, Ljiljana’s account demonstrates how ‘some women were able to adjust to capitalism using interpersonal, education, and material resources designed for survival under communism – a radically different social, political, and economic system’.^77^

Personal narratives of blue-collar workers illustrate the diverse ways in which they experienced socioeconomic dislocation in Serbia in the 1980s and 1990s demonstrating the extent to which subjective experience enabled the individual to adjust to radically altered conditions – or not. Ljiljana’s partner Nebojša had a somewhat different understanding of 1993. The pair did not become a couple until after 2000 and when discussing the 1990s they stressed the contrasting nature of their experiences. Nebojša was employed in the same firm as Ljiljana as a skilled metal-worker, with a higher income. He had built a comfortable family home on the peripheries of Belgrade following an eight-year stint aboard at steelworks in Germany in the late 1960s and early 1970s. Being some 15 years Ljiljana’s senior he was at a different life stage and did not have to worry about taking care of young children anymore – his family was already provided for. While Ljiljana recalled the enterprising ways in which she was able to earn money through informal practices, Nebojša chimed in expressing admiration for his partner, ‘I did not do that, nor would I ever be able to!’^78^ He lacked the skills of ‘making do’ that Ljiljana had acquired in the 1980s and instead he exchanged his foreign currency savings little by little and waited for the worst of the crisis to pass and for his wages to recover with the end of hyperinflation in 1994.

## The tolerable 1990s workplace (through the prism of an uncertain future)

The neoliberal economic restructuring of the economy after 2000 with the ousting of Milošević resulted in the closure of many factories in Serbia in the last two decades and an acceleration of deindustrialization which had already begun in the early 1990s. Economic recovery which occurred between the fall of Milošević in 2000 and the global crisis which enveloped Serbia in late 2008, resulted in wage increases and improved living standards. As Marek Mikuš points out, however, in a ‘context of deindustrialisation and jobless growth’ this benefited the middle classes rather than declassed workers as those employed in loss-making industries began to be laid off.^79^ Blue-collar workers were ‘left to the law of the market’ without many of the instruments of social protection they had been accustomed to in socialism and in a more limited way under the Milošević regime in the 1990s.^80^ Particularly since 2008, many workers and former workers are now objectively in a more difficult situation than they were in the 1990s. They suffer reduced access to welfare and healthcare, increased prices of consumer goods as well as the perception that they are worse off compared with individuals whose standard of living has modestly improved since 2000.^81^

In the Milošević era lay-offs were not permitted (although many workers were sent on leave with reduced pay).^82^ Between 1991 and 1993 Maria Stambolieva points out that public spending actually increased in Serbia as the regime attempted to buy social peace (parallel to the financing of wars in Croatia and Bosnia Herzegovina).^83^ After 2000 the unemployment rate rose sharply as the labour market was liberalized and Serbia reintegrated in to the global economy.^84^ Although the 1990s is commonly represented as a difficult period, one of great insecurity and risk, the memory of it is informed by a rather difficult present for many workers and former workers. Consequently, not all representations of the 1990s workplace are uniformly negative as Ognjen Kojanić gauges in his ethnography of railway workers in Zaječar, Serbia in 2014.^85^ Some individuals recall state subsidies keeping minimal production in their workplace and skeletal welfare arrangements afloat. Others claim the continuation of a sense of sociability which evaporated with deindustrialization after 2000. Murky privatizations and bankruptcies, years of unpaid wages and pension contributions by employers in the private sector, price increases, diminishing welfare and sustained economic crisis since 2008 render certain aspects of the 1990s workplace ever more appealing.

Mirjana, who left the ailing wood processing collective in Makiš to take up employment at the more profitable textile producer Kluz in 1985, believed that the firm successfully maintained a minimal standard for its workers during 1992 and 1993. She elaborates:

There was a larger pay in Kluz than in the wood processing collective, double the pay […] we worked for the foreign market, for Germans, ‘NikolaS’, ‘Boss’, for Italians, for foreigners […] In 1992 during inflation it became ever worse, the situation in the country was like that, but to tell you the truth during the time of inflation they were a good collective, there was not fights there, they distributed bread [….] During the time of inflation you could not come from work and find bread for sale. So we all got a loaf of bread to take home. Then in 1993 I remember we were getting various groceries – beans, sugar, potatoes, we got all of that… You know what, we didn’t feel inflation at all, because we got all of that from the firm, we brought it home and we had all we needed at home.^86^

In 1993 some firms like Kluz still adhered to elements of the socialist notion of citizenship where the workplace was at the ‘centre of one’s social universe’ with a major welfare function.^87^ A duty existed not only to pay a wage and make healthcare and pension contributions – the workplace would also ensure that foodstuffs in shortage could be accessed and so a minimal level of subsistence could be secured for workers like Mirjana and their families. Some elements of the corporatist Yugoslav vision of workplace identification continued into the 1990s (albeit in a limited way).

Mirjana’s younger brother Goran was employed as a security guard for Yugoslav furniture manufacturer Simpo since the closure of the wood processing plant in Makiš where he had worked like his parents and siblings. In April 2014 he believed Simpo to be on the verge of bankruptcy. Goran was struggling to make ends meet having not received wages for five months. His wife’s meagre salary as a post-office clerk was insufficient in providing for them and their two children. Having grown up in the workers’ settlement of Makiš during late socialism he associates it with poverty, describing the insufficient infrastructure – dilapidated barracks which had been converted into makeshift family homes – and meagre wages. However, upon further reflection he reconsiders, stating:

The wages were poor [during socialism and the 1990s] … wait, no, actually the wages were ok. We lived just ok [*onako*] but now, terribly. I work in Simpo now with no pay for months. Before it was great, there were 3,000 employees, three shifts, the canteen… you should have seen it. But now, what it looks like [… *Goran gestures towards the dilapidated warehouse building*].^88^

In describing Simpo, Goran’s narrative not only stresses the wages (which he states were ‘ok’) but a broader sense of sociality in which he invested pride (‘you should have seen it’). The Yugoslav factory was similar to the East German context as observed by Daphne Berdahl, in that ‘the workplace was not only the center of everyday sociality, it was a symbolic space of community and national belonging’.^89^ In contrast to this sense of sociality and community, Goran’s brother-in-law Milovan detailed the grim labour regimes that blue-collar workers faced, not only in Simpo but across the faltering or bankrupt firms of contemporary Serbia and other post-socialist states.

The younger workers have to get by somehow, they cannot retire as they are too young, say 55 […] they might only have 25 working years [of pension contributions] and so they work privately [in addition to Simpo] on river cafes [*splavovi*] as security guards, those kind of things. A carpenter might work like that privately in order to survive and wait for some kind of pension. It is difficult.^90^

Although social deprivation necessitated that workers moonlight during late socialism and in the 1990s, Goran stresses that those years were not that bad in comparison to the 2010s. ‘Simpo even exported during the time of Milošević under the director Tomić who was a SPS cadre.’^91^ Reflections like Goran’s should not necessarily be understood as an endorsement of the politics of Milošević but a reflection of the realities of deindustrializing post-2000 Serbia. The memory of more ample welfare, social and job security coupled with present day deprivation induces many narrators to (re)consider or gloss over past coercion. This is of course not specific to Serbia; in Romania for example David Kideckel observes that despite the brutal experience of late socialism under Ceausescu, (former) workers ‘readily downplay the state’s past coercion to look favourably on the security and enabling features of socialism’.^92^

Though characterized by hardship and war the 1990s in Serbia was nevertheless a time when a level of production occurred (however meagre) and workers could count on the legacy of the Yugoslav welfare state (however debilitated), particularly in cases where the director of the enterprise was close to the regime and could ensure production, the payment of a salary and the delivery of benefits-in-kind like groceries and other essential goods to stave off the worst effects of inflation, sanctions and the impoverishment of society. As Ivan Rajković details in his ethnographic study of the Zastava plant in Kragujevac, ‘buying social peace’ in this manner enabled workers to partake in smuggling other informal activities while keeping formal work status. Employment was less connected with productivity and increasingly ‘seen as a rent in the wider political system and a relative defence from the insecurities of the labour market’.^93^

The regulations of the neoliberalizing Serbian state in the mid-2010s are considered to be increasingly coercive towards the working class (particularly for the enterprising worker turned smuggler or petty trader) in denying them the means to ‘survive’. The state impinges on the moral right to subsistence which workers retrospectively consider they were able to access during the 1990s as employees in state firms. Opportunities to engage in the grey economy have been narrowed by the state which is considered by precarious narrators as increasingly coercive towards ordinary people in economic terms, for example with the introduction of fiscalization and increased inspections and fines for unlicensed petty trade which disproportionally affect feminized labour.^94^ Widespread unemployment not only threatens access to social welfare and pensions but inhibits certain economies of makeshift which had depended on employment status in a state industry directly or indirectly. As former anti-Milošević activist Nataša conceded, ‘at least during the time of Sloba [Slobodan Milošević] desperate people could set up a stall on ‘Bulevar’ [a main Belgrade thoroughfare and site of informal trade] and at have the chance to make ends meet. But now…’^95^

## Conclusion

This article has sought to highlight the malleability of memory and narrative in addressing continuities and ruptures as experienced by a small but diverse cohort of Belgrade based (former) blue-collar workers – at home, in the workplace and in their local community over the last few decades. Although historical and social anthropological scholarship has posited a rupture with the violent dissolution of the Yugoslav state and socialist system in 1991, through life history interviews I demonstrate that this rupture is understood by workers in quite varied ways and an inflexible ‘before/after’ framework is not always the most productive means to frame social phenomena, nor the ways in which they are understood by workers and former workers. To insist on the ubiquity of such a rupture can obscure the heterogeneous ways in which macro level events and processes were experienced by Belgraders and other (former) Yugoslavs in their everyday lives.

By linking discussions of the last decades, it becomes clear that many individuals casually frame and cognitively link their experience of late socialism with the post-socialist era and vice versa. Attributes and understandings of the 1980s, 1990s, 2000s and 2010s are frequently enmeshed and confused (rather than being compartmentalized into a dichotomous pre/post 1991 framework). Many Yugoslav workers recalled experiencing a destruction of sociability in their workplace and everyday life as early as the mid-1980s. Some attest that this experience equipped them to successfully navigate even deeper crisis during the 1990s. Contemporary economic crisis in Serbia and social dislocation associated with neoliberal economic reforms since 2000, also deeply shape working-class narratives of late socialism and the 1990s. For some workers the sociability associated with the socialist self-managing workplace remained palpable in a limited way during the 1990s. The difficultly of earning a living with dignity in contemporary Serbia for declassed workers induces many to regard the limited production and depleted welfare arrangements of the 1990s in a more positive light. In the wake of sustained economic crises and restructuring which has impoverished large swathes of Serbia’s former workers, memories of everyday life and labour under the rule of Milošević have been reassessed and some of the coercive aspects of the decade skirted over and an emphasis instead placed on the relative security and sociability of the era.

An approach which is sensitive to the social positioning of individuals (in terms of class, gender and age) and relies on experiential biographical narrative, reveals that a range of factors influenced how life trajectories before and after 1991 are represented. Class-informed experiences of privations in late socialist Yugoslavia exerted an influence on how individuals faced the social devastation of the 1990s as well as how they represent and recall ruptures associated with the multifaceted dissolution of Yugoslavia (the collapse of the socialist system and the outbreak(s) of war between 1991 and 1999). Notions of rupture in the life courses of workers have a rather stubborn longue durée and demand an appreciation for the post-socialist as well as post-conflict experience (albeit that these are muddled and intertwined).

Paying close attention to the diversity of life trajectories can give voice to narratives that go against the grain of conventional wisdom which assumes the experience of everyday life for ordinary people in Serbia during the 1990s to be universally negative and upward social mobility as the sole preserve of a criminal elite associated with the Milošević regime. For some, the break in the system in the early 1990s opened a space for entrepreneurial initiative and thus an improvement in their standard of living. Individuals like Ljiljana, who had toyed in the grey economy in the 1980s to supplement meagre factory earnings, quickly realized that the collapse of the system offered opportunity. For other workers, like Goran and his sister Mirjana, the 1990s did not necessarily offer obvious opportunity but the decade is remembered as a time when the faltering labour and welfare regimes were more secure and predictable for blue-collar workers than they are in the 2010s. Looking back at a difficult and contested recent past, workers and former workers in contemporary Serbia and other post-socialist countries are wont to paraphrase the graffito which opened this paper – ‘it was better when it was worse’.

## Disclosure statement

No potential conflict of interest was reported by the author.

## Funding

This work was supported by the Austrian Science Fund (FWF) [grant number P27008].

## Footnotes

^1^ I am very grateful to Tamara Buble for explaining the context of the comic series Maxmagnus. On Pula as an innovative site of recent developments in Yugoslav historiography see: A. Kladnik, ‘Socialism on the Bench’, *Social History*, 4, 3 (2016), 319–25.

^2^ S. Jansen, *Antinacionalizam: Etnografija otpora u Zagrebu i Beogradu* (Beograd, 2005); L. Spaskovska, ‘The Yugoslav chronotope: histories, memories and the future of Yugoslav Studies’, in F. Bieber, A. Galijas and R. Archer (eds), *Debating the End of Yugoslavia* (Farnham, 2014); J. Obradović-Wochnik, *Ethnic Conflict and War Crimes in the Balkans: the narratives of denial in post*-*conflict Serbia* (London, 2013), 102; A. Stefansson, ‘Urban exile: locals, newcomers and the cultural transformation of Sarajevo’ in X. Bougarel, E. Helms and G. Duijzings (eds), *The New Bosnian Mosaic: identities, memories and moral claims in a post*-*war society* (Aldershot, 2007).

^3^ Obradović-Wochnik, *op. cit.*, 66.

^4^ Spaskovska, *op. cit.*, 251.

^5^ On Yugoslavia’s dissolution see: F. Bieber, A. Galijaš and R. Archer (eds), *Debating the End of Yugoslavia* (Farnham, 2014); J. Lampe, *Yugoslavia as History: twice there was a country* (New York, 1996); B. Magaš, *The Destruction of Yugoslavia: tracing the break*-*up 1980*−*1989* (London, 1993); S. Ramet, *The Three Yugoslavias*: *state*-*building and legitimation, 1918*–*2005* (Washington DC, 2006).

^6^ E. Gordy, *The Culture of Power in Serbia: nationalism and the destruction of alternatives* (University Park PA, 1999), 165–98; R. Thomas, *The Politics of Serbia in the 1990s* (New York, 1999), 163–75; T. Judah, *The Serbs: history, myth and the destruction of Yugoslavia* (New Haven, 2000), 259–78.

^7^ Obradović-Wochnik, *op. cit*., 61–62.

^8^ M. Živković, *Serbian Dreambook: national imaginary in the time of Milošević* (Bloomington, 2011).

^9^ T. Petrović, ‘“When we were Europe”: socialist workers in Serbia and their nostalgic narratives’ in M. Todorova (ed.), *Remembering Communism: genres of representation* (New York, 2010), 128. See also O. Kojanić, ‘Nostalgia as a practice of the self in post-socialist Serbia’, *Canadian Slavonic Papers*, 57, 3–4 (2015), 195–212; T. Petrović, ‘The past that binds us: Yugonostalgia as the politics of the future’ in S. Pavlovic and M. Zivkovic (eds), *Transcending Fratricide: political mythologies, reconciliations, and the uncertain future in the former Yugoslavia* (Baden-Baden, 2013), 129–47.

^10^ T. Vetta, ‘Nationalism is back! Radikali and privatization processes in Serbia’ in D. Kalb and G. Halmai (eds), *Headlines of Nation, Subtexts of Class: working*-*class populism and the return of the repressed in neoliberal Europe* (New York, 2011).

^11^ P. Leavy, *Oral History: understanding qualitative research* (Oxford, 2011), 4; A. Portelli, ‘What makes oral history different’ in R. Perks and A. Thomson (eds), *The Oral History Reader* (London, 1998); L. Passerini, ‘Work ideology and consensus under Italian fascism’, *History Workshop*, 8, 1 (1979), 82–108.

^12^ Portelli, *op. cit*., 67.

^13^ Spaskovska, *op. cit.*, 241.

^14^ J. Dragović-Soso, *Saviors of the Nation: Serbia’s intellectual opposition and the revival of nationalism* (London, 2002); D. Jović, *Yugoslavia: a state that withered away* (West Lafayette, 2009); N. Vladisavljević, *Serbia’s Antibureaucratic Revolution: Milošević, the fall of communism and nationalist mobilization* (Basingstoke, 2008).

^15^ A. Magid, *Private Lives/Public Surfaces: grassroots perspectives and the legitimacy question in Yugoslav socialism* (Boulder, 1991). On textile workers see: C. Bonfiglioli, ‘Gendered citizenship in the global European periphery: textile workers in post-Yugoslav states’, *Women’s Studies International Forum*, 49 (2015), 57–65; C. Bonfiglioli, ‘Gender, labour and precarity in the South East European periphery: the case of textile workers in Štip’, *Contemporary Southeastern Europe*, 1, 2 (2014), 7–23; N. Vodopivec, ‘Yesterday’s heroes: spinning webs of memory in a postsocialist textile factory in Slovenia’ in D. Koleva (ed.), *Negotiating Normality: everyday lives in socialist institutions* (New Brunswick 2012); Petrović, ‘When We Were Europe’, *op. cit*.

^16^ But see: V. Arzenšek, ‘Radnički pokret i samoupravni socijalizam’, *Međunarodni radnički pokret* 29, 1-2 (1986), 56–67; V. Cvjetičanin, *Klasno biće jugoslovenskog društva* (Beograd, 1989); J. Obradović and W. Dunn (eds), *Workers, Self*-*Management and Organizational Power in Yugoslavia* (Pittsburgh,1978).

^17^ M. Westrate, *Living Soviet in Ukraine from Stalin to Maidan: under the falling red star in Kharkiv* (Lanham MA, 2016); M. Vanek, *Velvet Revolutions: an oral history of Czech society* (Oxford, 2016); D. Bertaux, P. Thompson and A. Rotkirch (eds), *On Living through Soviet Russia: living through the Soviet system* (New Brunswick NJ, 2005).

^18^ D. Raleigh, *Russia’s Sputnik Generation: soviet baby boomers talk about their lives* (Bloomington, 2006); D. Raleigh, *Soviet Baby Boomers: an oral history of Russia’s Cold War generation* (New York, 2012); P. Messana, *Soviet Communal Living: an oral history of the Kommunalka* (New York, 2011); J. Gheith and K. Jolluck, *Gulag Voices: oral histories of Soviet incarceration and exile* (New York, 2011).

^19^ M. Hájek and B. Vann, ‘Gendered biographies: the Czech state-socialist gender order in oral history interviews’, *Sociologicky Casopis/Czech Sociological Review*, 51, 6 (2015), 1077–104; K. Daskalova (ed.), *Voices of Their Own: oral history interviews of women* (Sofia, 2004); S. Penn and J. Massino (eds), *Gender Politics and Everyday Life in State Socialist Eastern and Central Europe* (Basingstoke: 2009). In a Yugoslav context see Bonfiglioli, ‘Gendered citizenship in the global European periphery’, *op. cit*.

^20^ N. Khanenko-Friesen and G. Grinchenko (eds), *Reclaiming the Personal: oral history in post*-*socialist Europe* (Toronto, 2015).

^21^ On labour history and archival research in a Yugoslav context see: R. Archer and G. Musić, ‘Approaching the socialist factory and its workforce: considerations from fieldwork in (former) Yugoslavia’, *Labor History*, 58, 1 (2017), 44–66; N. Baković, ‘Using local archives for a historical reevaluation of socialism. Examples of bankrupted factories’ collections and rehabilitation rrocesses in Čačak region (Serbia)’, *Revista Arhivelor* 2 (2011), 42–54; S. Rutar, ‘Towards a southeast European history of labour: examples from Yugoslavia’ in S. Rutar (ed.), *Beyond the Balkans: towards an inclusive history of southeastern Europe* (Vienna 2014); V. Vukliš, ‘Writing social history of socialist Yugoslavia: the archival perspective’, *Archival Science*, 17, 1 (2017), 55–77.

^22^ T. Lummis, ‘Structure and validity in oral evidence’ in R. Perks and A. Thompson (eds), *The Oral History Reader* (London, 1998), 274.

^23^ On housing and its relationship to society in Yugoslavia see: B. Le Normand, *Designing Tito’s Capital: urban planning, modernism, and socialism in Belgrade* (Pittsburgh, 2014); S. Vujović, ‘Društvene nejednakosti u stanovanju’ in M.V. Popović et al. (eds), *Društvene nejadnakosti* – *sociološko istraživanje u Beogradu* (Belgrade, 1987).

^24^ A. Simić, *The Peasant Urbanites: a study of rural*-*urban mobility in Serbia* (New York, 1973), 32–33.

^25^
*Statistički godišnjak Beograda 1989,* 27.

^26^ Z. Volčič, ‘Belgrade vs. Serbia: spatial re-configurations of belonging’, *Journal of Ethnic and Migration Studies*, 31, 4 (2005), 639–58; see also Jansen *Antinacionalizam*, *op. cit*.; S. Jansen, ‘Who’s afraid of white socks? Towards a critical understanding of post-Yugoslav urban self-perceptions’, *Ethnologia Balkanica*, 9 (2005), 151–67.

^27^ Simić, *op. cit*., 34.

^28^
*ibid.*

^29^ On the dilemmas of implicit discourses and insider/outsider-hood while conducting ethnography in contemporary Serbia see M. Simić, ‘Fieldwork dilemmas: problems of location, insiderhood, and implicit discourses’, *Гласник Етнографског института САНУ*, 58, 2 (2010), 26–39.

^30^ X. Bougarel, E. Helms and G. Duijzings (eds), *The New Bosnian Mosaic: identities, memories and moral claims in a post*-*war society* (Aldershot 2007); V.P. Gagnon, *The Myth of Ethnic War: Serbia and Croatia in the 1990s* (Ithaca 2004).

^31^ ‘Radničkoj klasi, radnim ljudima i građanima, narodima i narodnostima Socijalističke Federativne Republike Jugoslavije!’ *Ustav SFRJ*, 1974 cited in R. Archer, ‘Social inequalities and the Study of Yugoslavia’s dissolution’ in F. Bieber, A. Galijas and R. Archer (eds), *Debating the End of Yugoslavia* (Farnham, 2014), 136.

^32^ Archer, ‘Social inequalities’, *op. cit*.; see for example E. Berković, *Socijalne nejednakosti u Jugoslaviji* (Belgrade, 1986); Cvjetičanin, *Klasno biće, op.cit*.; R. Kilibarda, *Klasno biće Saveza komunista Crne Gore* (Titograd: 1984); V. Pešić, *Kratki kurs o jednakosti: Koncepcija jednakosti u zvaničnoj ideologiji Jugoslovenskog društva* (Belgrade, 1988); M. Popović et al. (eds), *Društveni slojevi i društvena svest* (Belgrade, 1977); M. Popović et al. (eds), *Društvene nejednakosti* – *sociološko istraživanje u Beogradu* (Belgrade, 1987).

^33^ A. Dević, ‘What nationalism has buried: Yugoslav social scientists on the crisis, grassroots powerlessness and Yugoslavism’ in R. Archer, I. Duda and P. Stubbs (eds) *Social Inequalities and Discontent in Yugoslav Socialism* (Abingdon, 2016), 24–25.

^34^ M. Lazić, *U susret zatvorenom društvu?* (Zagreb, 1987); S. Ramet, *Nationalism and Federalism in Yugoslavia 1962*–*1991,* (Bloomington, 1992).

^35^ I. Goldstein, *Croatia: a history* (London, 1999), 190; S. Woodward, *Socialist Unemployment: the political economy of Yugoslavia, 1945*–*1990*, (Princeton, 1995), 254–55.

^36^ Woodward, *Socialist Unemployment*, *op. cit*., 254.

^37^ Magaš, *op. cit*., 190.

^38^ Lampe, *op. cit*., 293; D. Bilandžić, *Jugoslavija poslije Tita 1980*–*1985* (Zagreb: Globus, 1986), 118–23.

^39^ W. Bartlett, ‘Foreign trade and stabilization policy in a self-managed economy: Yugoslavia in the 1980s’ in J. Allcock, J. Horton and M. Milivojević (eds), *Yugoslavia in Transition: choices and constraints* (Oxford/New York, 1991), 239; Bilandžić, *op. cit*.

^40^ C. Schierup, ‘Quasi-proletarians and a patriarchal bureaucracy: aspects of Yugoslavia’s re-peripherialization,’ *Soviet Studies*, 44, 1 (1992), 86.

^41^ P.H. Patterson, *Bought and Sold: living and losing the good life in socialist Yugoslavia* (Ithaca, 2011).

^42^ Magaš, *op. cit.*, 114n2.

^43^ S. Fočo, *Štrajk između iluzije i zbilje* (Belgrade, 1989).

^44^ B. Denitch, *Limits and Possibilities. The crises of Yugoslav socialism and state socialist systems* (Minneapolis, 1990) 69.

^45^ Gordy, *op. cit*.

^46^ Interview with Marija, Rakovica, Belgrade, March 2014.

^47^
*ibid.*

^48^ A.V. Ledeneva, *Russia’s Economy of Favours: Blat, networking and informal exchange* (Cambridge, 1998), 3.

^49^ E.P. Thompson, ‘The moral economy of the English crowd in the eighteenth century’, *Past & Present*, 50 (February 1971), 76–136; J. Scott, *The Moral Economy of the Peasant: rebellion and subsistence in Southeast Asia* (New Haven, 1976); M.A. Lebowitz, *The Contradictions of ‘Real Socialism’: the conductor and the conducted* (New York, 2012).

^50^ Lebowitz, *op. cit.,* 132; R. Archer, ‘The moral economy of home construction in late socialist Yugoslavia’, *History and Anthropology*, published online 26 Jun 2017, https://doi.org/10.1080/02757206.2017.1340279.

^51^
*ibid.*, 133, 139.

^52^ S. Cvek, S. Ivčić and J. Račić, ‘Jugoslavensko radništvo u tranziciji: “Borovo” 1989’, *Politička misao*, 52, 2 (2015), 7–34; G. Musić, ‘“They came as workers and left as Serbs”: the role of Rakovica’s blue-collar workers in Serbian social mobilizations of the late 1980s’ in R. Archer, I. Duda and P. Stubbs (eds), *Social Inequalities and Discontent in Yugoslav Socialism*, (Abingdon, 2016), 132–54; Vladisavljević, *op. cit.*

^53^
*ibid.*

^54^ Interview with Mirko, Makiš, Belgrade, April 2014

^55^ Interview with Milovan, Makiš, Belgrade, April 2014.

^56^ Interview with Mirjana, Makiš, Belgrade, April 2014.

^57^ Interview with Mirjana and Gordana, Makiš, Belgrade, April 2014.

^58^ Petrović, ‘When we were Europe’, *op. cit*., 128.

^59^ C. Baker, ‘Prosperity without security: the precarity of interpreters in postsocialist, postconflict Bosnia-Herzegovina’, *Slavic Review*, 71, 4 (2012), 849.

^60^ For example, see Gordy, *op. cit.*; Judah, *op. cit*.; Obradović-Wochnik, *op. cit.*

^61^ The relationship between silence, rumours and informal knowledge of wartime atrocities amongst Belgraders is detailed by Obradović-Wochnik, *op. cit.*, 102.

^62^ O.J. Schmitt, *Kurze Geschichte einer zentralbalkanischen Landschaft* (Köln 2008); M. Vickers, *Between Serb and Albanian: a history of Kosovo* (London, 1998).

^63^
*Kumstvo* refers to best-man and/or godfather-hood. Interview with Saša, Makiš, Belgrade, April 2014.

^64^ Cited in D.M. Hughes, L. Mladjenović and Z. Mršević, ‘Feminist resistance in Serbia’, *European Journal of Women’s Studies*, 2, 4 (1995), 509–32, 523.

^65^ According to trade union surveys: Magaš, *op. cit*., 98.

^66^ O. Hufton, *The Poor of Eighteenth*-*Century France* (Oxford, 1974).

^67^ S. Williams, *Poverty, Gender and Life*-*Cycle under the English Poor Law, 1760*–*1834* (Woodbridge, 2011); S. King and A. Tomkins (eds), *The Poor in England, 1700*–*1850: an economy of makeshifts* (Manchester, 2003); K. Hopper, E. Susser and S. Conover, ‘Economies of makeshift: deindustrialization and homelessness in New York City’, *Urban Anthropology and Studies of Cultural Systems and World Economic Development*, 14, 1/3 (1985), 183–236.

^68^ M. Stambolieva, *Welfare State Transformation in The Yugoslav Successor States: from social to unequal* (Abdingdon, 2016), 50–52.

^69^ Interview with Ljiljana, Novi Beograd, Belgrade, February 2014.

^70^ On accounts of shopping and petty smuggling from Trieste to Yugoslavia see M. Mikula, ‘Highways of desire: cross-border shopping in former Yugoslavia, 1960s–1980s’ in H. Grandits and K. Taylor (eds), *Yugoslavia’s Sunny Side. A History of Tourism in Socialism (1950s*–*1980s)* (Budapest, 2010).

^71^ P.T. ‘Smešno ćoše’, *Informator DMD*, br. 127, 29.02.1980, str.7.

^72^ Magaš, *op. cit*., 131.

^73^ Interview with Ljiljana (Novi Beograd), *op. cit*.

^74^ R. Archer and K. Rácz, ‘*Šverc* and the *Šinobus*: small-scale smuggling in Vojvodina’ in B. Bruns and J. Miggelbrink (eds), *Subverting Borders: doing research on smuggling and small*-*scale trade* (Wiesbaden, 2012)*,* 62.

^75^ P. Andreas ‘Criminalizing consequences of sanctions: embargo busting and its legacy’, *International Studies Quarterly*, 49 (2005), 335–60, 343.

^76^ Interview with Ljiljana (Novi Beograd), *op. cit*.

^77^ K. Ghodsee, *The Red Riviera: gender, tourism and postsocialism on the Black Sea* (Durham, 2005), 5–6.

^78^ Interview with Nebojša, Novi Beograd, Belgrade, February 2014.

^79^ M. Mikuš, ‘The justice of neoliberalism: moral ideology and redistributive politics of public-sector retrenchment in Serbia’, *Social Anthropology/Anthropologie Sociale*, 24, 2 (2016), 211–27, 220; see also M. Arandarenko, ‘Politička ekonomija nezaposlenosti’ in M. Arandarenko, A. Praščević and S. Cvejić (eds), *Ekonomsko*-*socijalna struktura Srbije: učinak prve decenije tranzicije* (Belgrade: 2010).

^80^ M. Lazić, *Čekajući kapitalizam* (Belgrade, 2011), 154–55; Stambolieva, *op. cit*., 158–59.

^81^ See G. Musić, *Serbia’s Working Class in Transition 1988*–*2013* (Belgrade 2013), 12–13; compare with M. Uvalić, *Serbia’s Transition: towards a better future* (London, 2010).

^82^ Stambolieva, *op. cit*., 158–59.

^83^
*ibid.*

^84^
*ibid.*, 162.

^85^ Kojanić, *op. cit*., 196.

^86^ Interview with Mirjana (Makiš),*op. cit*.

^87^ S. Woodward, ‘The political economy of ethno-nationalism in Yugoslavia’, *Socialist Register*, 39 (2003), 73–92, 76.

^88^ Interview with Goran, Makiš, Belgrade, April 2014.

^89^ D. Berdahl, ‘(N)Ostalgie’ for the present: memory, longing, and East German things’, *Ethnos*, 64, 2 (1999), 194.

^90^ Interview with Milovan (Makiš), *op. cit*.

^91^ Interview with Goran (Makiš), *op. cit*. SPS (Socialist Party of Serbia) was the ruling party during the 1990s under the tutelage of Slobodan Milošević.

^92^ D. Kideckel, *Getting by in Postsocialist Romania: labor, the body and working*-*class culture* (Bloomington, 2008), 17–18.

^93^ I. Rajković, ‘“Labour now! Tracing industrial slowdown, (un)productive employment and calls for work ethic in postsocialist Serbia’, paper presented at ‘Socialism on the Bench’ conference, Pula, 1–3 October 2015.

^94^ For example, S. Dragojlo, ‘Serbia toughens inspections of “grey economy”, *Balkan Insight* http://www.balkaninsight.com/en/article/new-grey-economy-inspection-law-against-poorest (accessed 25 August 2015).

^95^ In conversation with the author, October 2013.

